# Emergence of Unusual G6P[6] Rotaviruses in Children, Burkina Faso, 2009–2010

**DOI:** 10.3201/eid1804.110973

**Published:** 2012-04

**Authors:** Johan Nordgren, Leon W. Nitiema, Sumit Sharma, Djeneba Ouermi, Alfred S. Traore, Jacques Simpore, Lennart Svensson

**Affiliations:** Linköping University, Linköping, Sweden (J. Nordgren, S. Sharma, L. Svensson);; Université de Ouagadougou, Ouagadougou, Burkina Faso (L.W. Nitiema, D. Ouermi, A.S. Traore, J. Simpore)

**Keywords:** rotavirus, G6P[6] rotaviruses, genotypes, G genotype, P genotype, acute gastroenteritis, emergence, children, Burkina Faso, surveillance, viruses

## Abstract

To obtain more information about rotavirus (ROTAV) genotypes in Burkina Faso, we characterized 100 ROTAVs isolated from fecal samples of children with acute gastroenteritis in the capital city of Ouagadougou, during December 2009–March 2010. Of note, 13% of the ROTAV-positive samples, including those with mixed infections, were positive for the unusual G6 genotype ROTAV strain. The genotypes identified were G9P[8], G6P[6], G1P[6], G3P[6], G1P[8], and G2P[4]. G9P[8] subgroup (SG)II strains dominated during the beginning of the ROTAV season, but later in the season, other G types associated with P[6] and SGI specificity emerged. This emergence was related to a shift in the overall age of infected children; ROTAV SGII infected younger children and induced more severe symptoms. The finding of a high incidence of G6P[6] strains highlights the need for long-term surveillance of ROTAV strains in Burkina Faso, especially when ROTAV vaccination is being considered in several African countries.

## Abstract 2

High incidence highlights the need for long-term surveillance of rotavirus strains.

Rotavirus (ROTAV) is a leading cause of severe acute gastroenteritis in infants and young children (*1*). It is estimated that ROTAV is responsible for 527,000 deaths each year; most occur in children from developing countries (*2*). More than 230,000 of these deaths occur in sub-Saharan Africa, and 6 of the 7 countries for which ROTAV diarrhea–related mortality rates are highest are located in Africa (*2*).

The viral protein (VP) 7 and VP4 genes encoding the 2 surface proteins form the basis of the current genotypic characterization of group A ROTAVs into G and P genotypes, respectively. At least 27 G genotypes and 35 P genotypes have been identified (*3*). Of these, 6 ROTAV G-P combinations (G1P[8], G2P[4], G3P[8], G4P[8], G9P[8], and G9P[6]) are common worldwide (*4**–**6*); several other combinations occur at low frequencies, and a few others occur mainly in animals (*7**,**8*). On the basis of molecular and immunological analysis, the VP6 gene has been classified into 2 subgroups (SGI and SGII); SGII is the most prevalent among humans (*9**,**10*) and SGI is more commonly found in animals (*11*).

The G-P combinations circulating in Africa often differ from those circulating in other parts of the world (*12*). A review of studies in Africa published during 1997–2006 showed that the most common G and P type combinations were G1P[8] (17.4%), G2P[6] (9.6%), G8P[6] (9.4%), and G3P[8] (7%) (*13*). A study from West Africa, covering 1996–2000, found that G1P[6] was the most common G-P combination, followed by G2P[6] and G9P[8] (*12*). During 1998–2000, the G9P[8] combination was the most prevalent genotype in northern Ghana (*14*). However, many ROTAV strains isolated in Africa still remain to be characterized for G and/or P type (*13*).

Although bovine G6 ROTAV strains are the predominant genotype in cattle in Africa, G6 infection in humans has rarely been described; however, sporadic cases of G6 in humans in combination with P[14] and P[9] have been described (*15**–**21*). P[6] is a commonly observed P type, especially in Africa. However, to our knowledge, the literature reports only once the isolation of strain G6P[6] in Belgium, from a child returning from a trip to Mali (*22*).

In Burkina Faso, previous studies have shown ROTAV to be the main cause of diarrhea in children (*23**–**26*), but limited molecular information exists about the circulating genotypes. The first molecular characterization of ROTAVs, which was conducted on samples from 1999, showed a predominance of unusual G2P[6]SGI strains (*27*).

In this study, we determined the G and P genotypes of ROTAVs isolated from children with acute gastroenteritis in Ouagadougou, the capital of Burkina Faso, during the cold, dry season of December 2009–March 2010. We found that G6P[6] ROTAV strains were the second most common genotype circulating; incidence was 13%. We discuss the emergence of this most unusual genotype in association with vaccine efficacy and zoonotic transfer.

## Materials and Methods

### Study Population and Specimens

Fecal specimens were collected from children <5 years of age who sought medical care for acute diarrheal illness, defined as >3 liquid stools over a 24-h period. Stool specimens were collected in sterile containers at the microbiology laboratory in Saint Camille Medical Center, in Ouagadougou, Burkina Faso. A 10% (wt/vol) stool suspension was prepared, and 3 aliquots were frozen at −20°C for additional analysis. All ROTAV-positive fecal specimens collected during the ROTAV season (December 2009–March 2010) at the Saint Camille Medical Center and the Biomolecular Research Center Pietro Annigoni, were analyzed. The samples were part of a larger epidemiologic study conducted during May 2009–March 2010 to identify the enteropathogens causing gastroenteritis in children (*26*).

### Clinical Assessment

Clinical information was obtained by reviewing the clinical records of case-patients, as described (*26*). In brief, information was obtained regarding age, sex, place of residence, ethnicity, signs and symptoms and their duration (e.g., fever [temperature >38°C], nausea, vomiting, loss of appetite, and number of loose stools during the past 24 h), hydration and nutrition status, and whether the children had been given antimicrobial and antiparasitic drugs. All children were clinically evaluated by general practitioners in accordance with a local adaption of the World Health Organization strategy for diarrheal management (*28*). Dehydration was classified as follows, according to the World Health Organization guidelines: severe dehydration, some dehydration, or no dehydration.

### Rotavirus Antigen Detection

We used the ProSpect Rotavirus R240396 (Oxoid, Kamstrupvej, Denmark) enzyme immunoassay kit, according to the manufacturer’s instructions, to detect group A human ROTAV in fecal specimens. The results were determined visually and confirmed by absorbance readings.

### Viral RNA Extraction

Viral RNA was extracted from 10% stool suspensions by using the QIAamp Viral RNA Mini Kit (Qiagen, Hilden, Germany) according to the manufacturer’s instructions. A total of 60 μL of viral RNA was collected and stored at −70°C until further use.

### Reverse Transcription

Reverse transcription (RT) was performed essentially as described (*29*). Twenty-eight μL of dsRNA was mixed with 2.5 μg of random hexadeoxynucleotides (pd[N]_6_ primer; GE Healthcare, Uppsala, Sweden), denatured at 97°C for 5 min, and chilled on ice for 2 min. The suspension was then added to 1 RT-PCR bead (GE Healthcare) with RNase-free water to a final volume of 50 μL. The RT reaction was performed for 30 min at 42°C for cDNA synthesis.

### Quantification and VP6 Subgrouping by Real-time PCR

All ROTAV-positive specimens were quantified and subgrouped by using the LUX (light-upon-extension) real-time quantitative PCR (qPCR) as described (*30*). This qPCR uses labeled primers with different fluorophores for each VP6 subgroup, and external plasmid standards were used for quantification (*30*). The quantified ROTAV was detected in a range from ≈5.0 × 10^4^ to 7 × 10^11^ gene equivalents per gram of feces; median concentration was 7.8 × 10^9^.

### G and P Typing

G and P genotyping for ROTAV was performed by using seminested type-specific multiplex PCRs that were able to detect 7 G types (G1, G2, G3, G4, G8, G9, G10) and 6 P types (P[4], P[6], P[8], P[9], P[10], P[11]) (*31**–**33*). The PCR products were later visually examined on a 2% agarose gel stained with ethidium bromide and observed in ultraviolet light. The G and P types were determined by the specific sizes of the amplicons on agarose gels.

### Sequencing of Untyped Strains and Molecular Characterization of G6P[6] Strains

Sequencing of the PCR amplicons for the VP7 gene of unknown G types was conducted with primers VP7-F and VP7-R (*33*). All G6P[6] strains were further sequenced for VP4 and VP6 genes by using primers Con-2/Con-3 (*32*) and GEN_VP6F/GEN_VP6R (*7*), respectively. Furthermore, to understand the evolution of these G6P[6] ROTAV strains, we randomly selected 4 strains and sequenced the nonstructural protein (NSP) genes (NSP1, NSP2, NSP3, NSP4, and NSP5) by using primers GEN_NSP1F/GEN_NSP1R, MAX-NSP2F/ MAX-NSP2R, MAX-NSP3F/MAX-NSP3R, GEN_NSP4F/GEN_NSP4R, and MAX-11F/ MAX-11R, respectively (*7*). The following thermal cycling conditions were used: an initial denaturation step at 94°C for 5 min followed by 40 cycles of amplification (30 s at 94°C, 30 s at 50°C, and 1 min 30 s at 72°C), with a final extension of 7 min at 72°C.

### Nucleotide Sequencing

Nucleotide sequencing was performed by Macrogen Inc. (Seoul, South Korea). The sequencing reaction was based on BigDye chemistry, and the sequencing primers were the same primers as those used in the PCR.

### Sequence Analysis of Mixed Infections

All chromatograms were visually inspected. Those containing multiple ambiguous base positions were considered to potentially represent multiple ROTAV strains and were analyzed by using the RipSeq mixed DNA interpretation software (iSentio Ltd., Bergen, Norway) (*34*).

### Sequence Analysis

Multiple sequence alignment of the obtained nucleotide sequences was performed by using the ClustalW algorithm (www.ebi.ac.uk/Tools/msa/clustalw2/) with default parameters on the European Bioinformatics Institute server. Phylogenetic analysis of the aligned file was performed by using MEGA5 (www.megasoftware.net) with the neighbor-joining method. Phylogenetic distances were measured by using the Kimura 2-parameter model. The statistical significance of the phylogenetic tree was supported by bootstrapping with 1,000 replicates. The sequenced ROTAV strains and GenBank accession numbers are shown in Technical Appendix Table 1; the nucleotide sequences for sequenced genes can be found in GenBank by using accession nos. JN116505-JN116556 and JQ255029-JQ255033.

### Statistical Analysis

Categorical data were analyzed by using the χ^2^ test or Fisher exact test with 2-tailed significance. Interval data were analyzed by running an independent *t*-test with 2-tailed significance in SPSS version 17.0 (SPSS Inc., Chicago, IL, USA).

## Results

### High incidence of the Unusual G6P[6] Genotype

The distribution of G and P types is shown in Table 1. We sequenced all unknown G types for which the VP7 amplicon could be generated and found that they belong to genotype G6 with P[6] specificity. Of the 100 ROTAV-positive specimens, 13 contained G6P[6], including 2 with mixed infections with other G types. We detected 5 G types: G1 (23% incidence rate), G2 (2%), G3 (9%), G6 (13%), and G9 (53%). Among the P genotypes, P[8] was most prevalent (61%), followed by P[6] (39%); P[4] was rarely detected (2%). The G-P combinations detected were G9P[8] (48%), G6P[6] (11%), G1P[6] (11%), G3P[6] (8%), G1P[8] (5%), and G2P[4] (2%). Mixed G or P type infections were found in 9% of the total specimens. Among the ROTAV-positive specimens, 6% and 1% could not be assigned a G and P type, respectively (Table 1).

**Table 1:** Distribution of G and P types of rotavirus strains detected among children with gastroenteritis, Ouagadougou, Burkina Faso, December 2009–March 2010

Rotavirus type	No. (%) strains					
	P[4]	P[6]	P[8]	P[mix]*	P[nt]	Total
G1	0	11	5	2	0	18 (18)
G2	2	0	0	0	0	2 (2)
G3	0	8	0	0	0	8 (8)
G6	0	11	0	0	0	11 (11)
G9	0	0	48	0	1	49 (49)
Gmix†	0	3	3	0	0	6 (6)
Gnt‡	0	3	2	1	0	6 (6)
Total	2 (2)	36 (36)	58 (58)	3 (3)	1 (1)	100 (100)

*P[6]/P[8]. †G1/G9P[6], G1/G6P[6], and G3/G6P[6] specificity was detected in 1 specimen each; 3 samples were found to be G1/G9P[8] specific. ‡Nontypeable.

### Seasonal Distribution of Circulating ROTAV Strains

**Figure 1:**
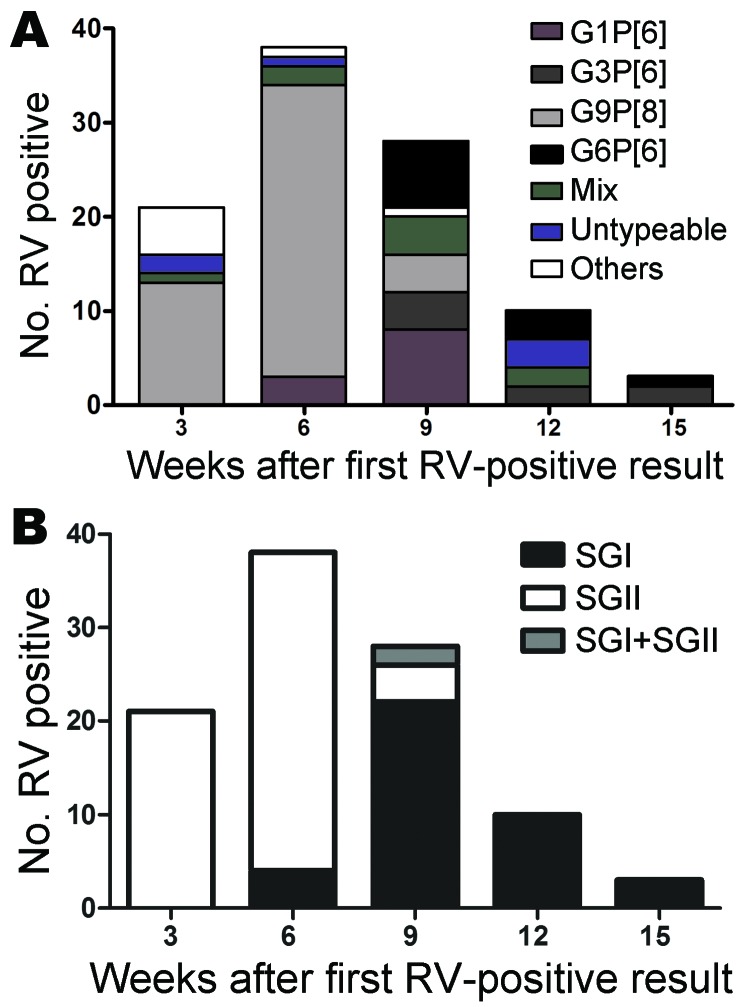
Temporal changes of circulating rotaviruses (ROTAVs) in Burkina Faso during December 2009–March 2010. A) G and P types, with “Others” representing G1P[8] and G2P[4]. B) Viral protein 6 subgroups. RV, rotavirus.

In the first part of the ROTAV season (December 2009 and early January 2010), we observed a dominance of G9P[8] strains (Figure 1, panel A). Thereafter, the G9P[8] strains completely disappeared and other G types began to circulate (Figure 1, panel A). All G types observed during this period were associated with P[6] genotype. Moreover, at the end of the ROTAV season, the G6P[6] and G3P[6] strains emerged, and most of the mixed infections were detected during this period. Furthermore, we observed a temporal shift in subgroup specificity (Figure 1, panel B). We subgrouped the VP6 gene by using our qPCR assay, and of the 100 ROTAV-positive specimens, 39 belonged to SGI, 59 belonged to SGII, and 2 specimens contained a mix of both subgroups. In the middle of the ROTAV season, we observed a shift of subgroups, with SGI dominating toward the end of the ROTAV season and SGII dominating in the beginning (Figure 1, panel B).

### SGII ROTAVs More Prevalent in Younger Children and Associated with More Severe Symptoms

All P[8] ROTAVs were associated with VP6 SGII, and all P[6] ROTAVs were associated with VP6 SGI. Children infected with ROTAV SGII had a mean age of 11.8 months (median 11.1 months), compared with a mean age of 15.0 months (median 13.0 months) for children infected with ROTAV SGI (p < 0.05). These observations correlate with the shift of VP6 subgroups and P types in the middle of the ROTAV season; children infected later were older than those who were infected early (data not shown). We compared clinical signs and symptoms and found no significant differences, but we did find higher prevalence of vomiting (p = 0.33), fever (p = 0.052), and severe dehydration (p = 0.09) among SGII-infected children (Table 2). We compared the 3 most prevalent G, P, and VP6 constellations (Table 2) and found no significant differences in age profiles; however, we found that ROTAVs of the G9P[8]SGII constellation were more frequent than G1P[6]SGI- and G6P[6]SGI-specific strains among younger children (p = 0.069). We also found that G9P[8]SGII infections, compared with SGI-associated G and P type infections, induced significantly more cases of severe dehydration (p<0.05). The ROTAV viral load, quantified by using the LUX qPCR, did not correlate with disease severity (data not shown).

**Table 2:** Clinical differences between subgroup SGI and SGII rotaviruses and the 3 most common G, P, and VP6 type constellations in Burkina Faso, December 2009–March 2010

Isolate type	No. specimens*	Patient characteristics and clinical signs										
		Age			Vomiting			Fever†			Severe dehydration	
		Mo ± SD	p value		No. (%)	p value		No. (%)	p value		No. (%)	p value
SGI	39	15.0 ± 8.5			30 (77)			14 (36)			5 (13)	
SGII	59	11.8 ± 5.0	0.038‡		50 (85)	0.33‡		33 (56)	0.052‡		16 (27)	0.09‡
G1P[6]SGI	11	15.5 ± 11.0			8 (73)			4 (36)			0 (0)	
G6P[6]SGI	11	16.6 ± 10.2			9 (82)			4 (36)			2 (18)	
G9P[8]SGII	48	11.6 ± 4.6	0.069§		43 (90)	0.320§		24 (50)	0.29§		16 (33)	0.03§

*Two samples positive for both SGI and SGII were excluded. †Temperature >38°C. ‡SGI versus SGII. §G9P[8]SGII versus G1P[6]SGI and G6P[6]SGI.

### Nonspecific Binding of the G9 Genotype Primer to a New Sublineage of G3 Strains

**Figure 2:**
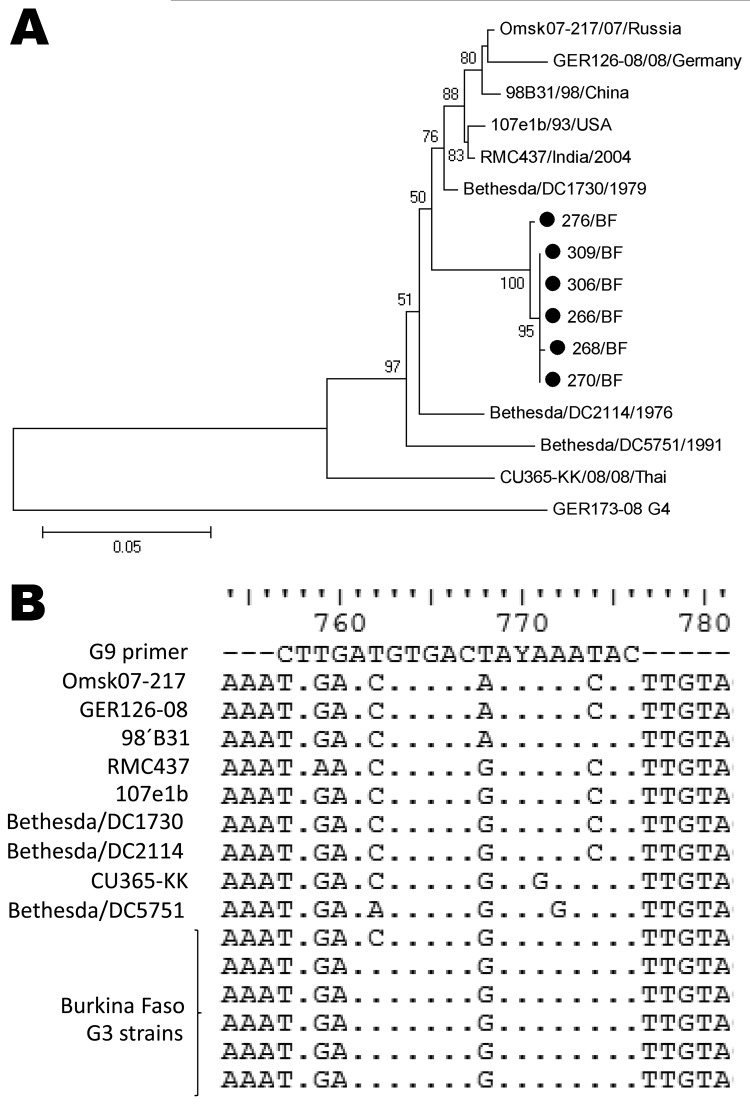
A) Phylogenetic analysis of partial sequences of the viral protein (VP) 7 genes (nt 115–851) for G3 rotavirus strains isolated in Burkina Faso during December 2009–March 2010, with a G4 strain as an outgroup. Scale bar represents number of substitutions per site. Bootstrap values are shown at the branch points (values of <50% are not shown). B) The G9 primer used is homologous at the 3′ end to the VP7 gene of the G3 strains in Burkina Faso during December 2009–March 2010. The reference G3 strains were deliberately selected for similarity to the Burkina Faso G3 strains.

At first, we observed a high number of G3/G9P[6] mixed infections as revealed by multiple bands in the multiplex PCR (data not shown). We sequenced the VP7 gene of these strains (n = 6) and found all 6 to have high nucleotide homology with G3 ROTAVs and to cluster together in a new sublineage (Figure 2, panel A). This sublineage had 96.0%–96.3% nt homology to the most similar G3 strain found in GenBank (Bethesda/DC1730). Furthermore, the G9 primer used in this study (*33*) was highly homologous (16 [80%] of 20 nt) to these G3 strains but less so to other G3 strains available in GenBank (maximum 14 [70%] of 20 nt) (Figure 2, panel B). Furthermore, the 14 bases from the 3′ end of the primer showed a complete match to the G3 strains from Burkina Faso, except at position 9, thereby facilitating unspecific primer annealing and polymerase extension. The VP7 gene of 6 G9P[8] samples was sequenced for verification which proved them to be G9. These findings led us to conclude that G9 ROTAVs appearing as mixed infections with G3P[6], as determined by multiplex G-typing PCR, were nonspecific amplifications resulting in G9 artifact.

### Molecular Characterization of the Rare G6P[6] Strains

**Figure 3:**
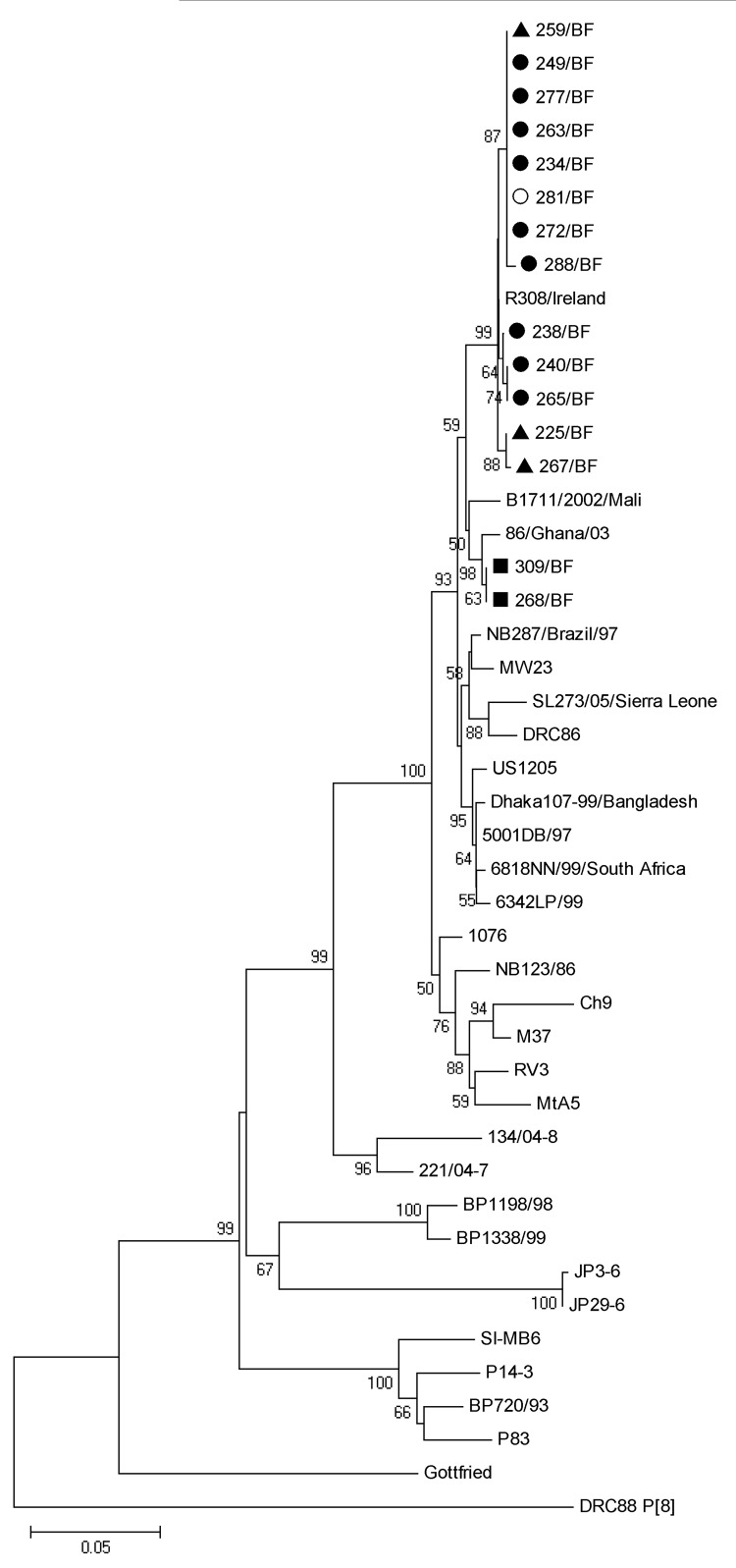
Phylogenetic analysis of partial sequences of viral protein (VP) 8 subunits of the VP4 gene of rotavirus (nt 141–751), with reference strains from all P[6] lineages and with the P[8] DRC88 strain as an outgroup. GenBank accession numbers for VP4 genes of reference strains are available in Technical Appendix Table 3. Filled circles, G6P[6] rotavirus strains; triangles, G1P[6] strains; squares, G3P[6] strains; open circles, G1G6P[6] strains from Burkina Faso, December 2009–March 2010. Scale bar represents the number of substitutions per site. Bootstrap values are shown at branch nodes (values of <50% are not shown).

**Figure 4:**
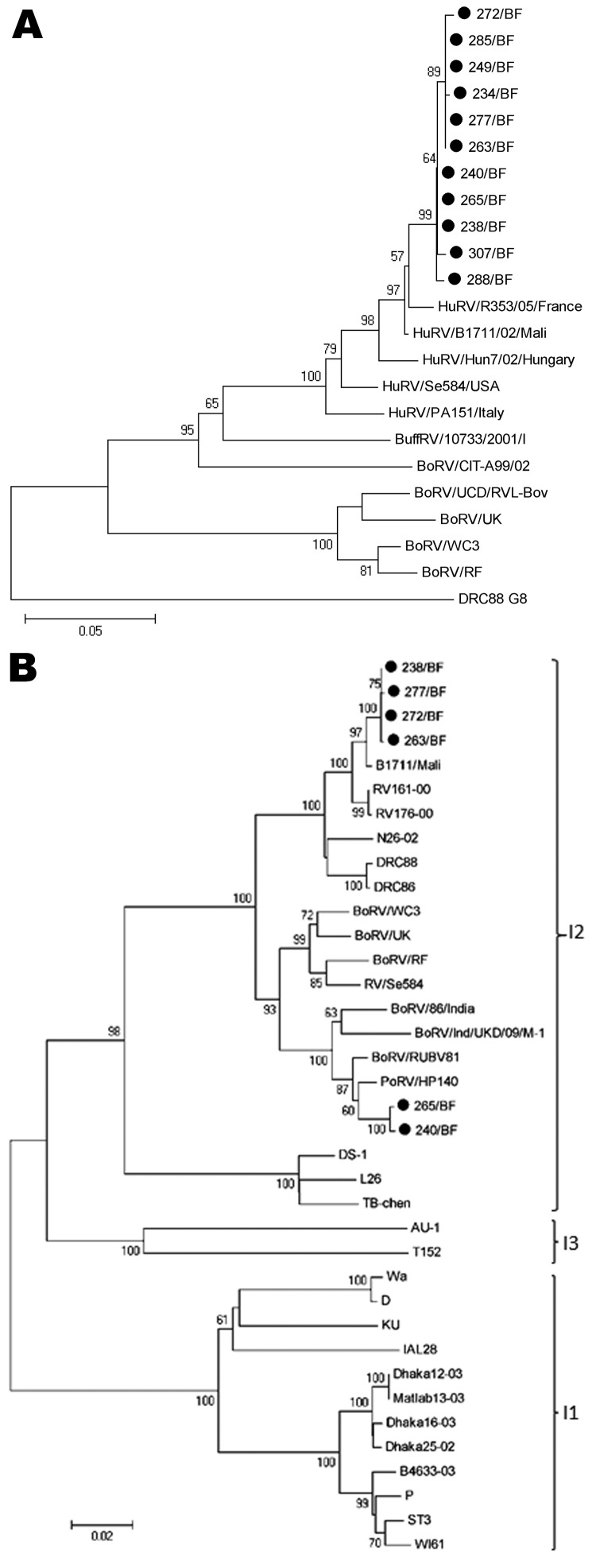
Phylogenetic analysis of partial sequences of A) viral protein (VP) 7 genes of the Burkina Faso G6P[6] rotavirus (ROTAV) strains (nt 135–796), with G8 strain DRC88 as an outgroup. GenBank accession numbers for VP7 genes of reference strains are available in Technical Appendix Table 4. B) VP6 genes (nt 145–1217) of the G6P[6] ROTAV strains from Burkina Faso, with I2 genotype specificity and reference strains from genotypes I1, I2, and I3. GenBank accession numbers. for VP6 genes of reference strains are available in Technical Appendix Table 5. Filled circles, Burkina Faso G6P[6] ROTAV strains. Bo, bovine; Buff, buffalo; Hu, human. Po, porcine. Scale bars represent number of substitutions per site. Bootstrap values are shown at the branch nodes (values of <50% are not shown).

We further characterized the unusual G6P[6] strains (n = 11) by sequencing the VP7, VP4, VP6, and NSP1–5 genes. All characterized VP4 and VP7 sequences clustered together, demonstrating high homology (Figure 3; Figure 4, panel A). The VP6 genes clustered in different lineages; 4 strains clustered with the B1711 strain, which is the only previously described human ROTAV G6P[6] strain (*22*), and 2 strains clustered closely with bovine strains (Figure 4, panel B). Phylogenetic analysis of the VP6 genes revealed that all strains clustered within lineage I2, which is typical of strains belonging to the DS-1 genogroup (*35*). However, the nucleotide difference between the 2 VP6 clusters was 7.7%–7.8%, strongly indicating different origins of the VP6 genes. To further characterize the G6P[6] strains from Burkina Faso, we sequenced the NSP1–5 genes for 4 of the G6P[6] strains (>80% nt coverage). All NSP genes of these 4 strains were highly homologous (>98.6% nt similarity); thus, we chose a representative isolate (272/BF) for comparison with reference strains (comparison results are shown in Technical Appendix Table 2).

The representative isolate, 272/BF, had a high level of identity with G6 (B1711) and G8 (DRC88) strains belonging to the DS-1 genogroup. The G6P[6] strains were classified as G6-P[6]-I2-A2-N2-T2-E2-H2, strongly suggesting that G6P[6] strains from Burkina Faso belong to the human DS-1 genogroup.

## Discussion

We characterized ROTAV strains circulating in Ouagadougou, Burkina Faso, during the cold, dry season (December 2009–March 2010). The strains were assigned a G and P genotype after multiplex RT-PCR or nucleotide sequencing and were subgrouped and quantified by using qPCR. This high incidence (13%) of the rare G6P[6] strain in humans during a ROTAV season is remarkable.

The circulating G and P genotypes showed a dynamic pattern, with G9P[8] strains dominating early in the cold season and then being replaced with various G types, mostly associated with the P[6] genotype. Using our VP6 subgroup-specific qPCR (*30*), we subgrouped all 100 ROTAV-positive specimens. All SGI ROTAV strains were associated with the P[6] or P[4] genotype, and all SGII ROTAV strains were associated with P[8], demonstrating a gene-dependent cosegregation pattern. In the middle of the season, there was a shift from SGII to SGI ROTAV strains, which also corresponded with a slightly older age group of infected children. Herd immunity could be an underlying factor for the differences in age profiles; SGI strains infected older children who may have been immunologically naive to ROTAV SGI but not SGII strains (*36*). Indeed, 3 of 4 children >2.5 years of age were infected with SGI-specific ROTAV strains, and the fourth child was infected with SGI and SGII.

Moreover, we observed that signs and symptoms induced by SGII infections were more severe than those induced by SGI infections; the ROTAV constellation G9P[8]SGII resulted in most cases of severe dehydration (33%). One possible explanation could be that children infected with SGI ROTAV were generally older and thus had an immune memory response from a previous ROTAV infection. It has been reported that children previously infected with ROTAV are protected against severe ROTAV-induced diarrhea during subsequent infections (*37*). Earlier studies also found differences in clinical manifestations between SGI and SGII infections (*36**,**38*). The age-group profile for the SGI- and SGII-infected children in those studies also differed; thus, it is plausible that results from those studies could also be associated with age differences for children infected with the different subgroups.

The G9 primer was highly homologous to the Burkina Faso G3 ROTAV sublineage. This homology demonstrates the need for continually updating the PCR methods used for assessing G and P type specificity and points out the need for caution when using multiplex PCRs, which could lead to an overestimation of the prevalence of mixed infections or inaccurate genotype assessment (*39**,**40*). In this study, a random selection from each G-P combination was sequenced to verify correct assignment as determined by the multiplex PCR assay.

The major finding of this study was the high proportion (13%) of G6P[6] strains circulating in Ouagadougou, Burkina Faso. A study from Belgium reported the isolation of a G6P[6] strain from a child returning from Mali (*22*), which to our knowledge is the only description published to date of a human infection with G6P[6]. Many ROTAV strains from sub-Saharan Africa remain untypeable, and the high incidence of G6P[6] observed in this study suggests that G6P[6] ROTAV strains might be more widespread than earlier thought. If this was an atypical event in Burkina Faso, or if the G6P[6] strains are more widespread in Africa than previously assumed, more untypeable strains from the region should be investigated by sequencing of the relevant genes. G6 ROTAVs are the most prevalent genotype in cattle worldwide (*8*), but they are rarely found in humans. The high number of G6P[6] sequences found in our study and the fact that all the G6 sequences clustered with the human G6P[6] reported (B1711) indicates a recent introduction. The VP4 gene of the G6 strains was highly homologous to the human B1711 strain and also clustered with a human P[6] strain detected in Ireland (R308). Because bovine G6 strains are highly prevalent in cattle, it is possible that the VP7 gene found in this study is derived from a transmission event between cattle and humans. In the peripheral areas of Ouagadougou, as well as Burkina Faso in general, cattle and humans live in close proximity, thus increasing the possibility of ROTAV transmission between animals and humans. Also, human P[6] ROTAVs are common in Africa (*13*), suggesting that reassortment between a bovine G6 and human P[6] during co-infection could occur, as previously suggested (*22*). We also found 2 cases of mixed infection with G6, 1 with G1, and 1with G3, suggesting that reassortment with the G6 genotype might occur.

We further investigated the VP4 gene of cocirculating G1P[6], G3P[6], and G1/G6P[6] strains. We observed that the VP4 genes of the G3P[6] strains clustered separately as compared with the VP4 gene of the G6P[6] strains (97.5%–97.9% nt identity), whereas the VP4 genes of the G1P[6] and G1G6P[6] were highly similar to the VP4 gene of the G6P[6] strains (99%–100% nt identity), indicating reassortment between the G1P[6] and G6P[6] strains.

Nucleotide sequencing data of the VP6 gene for 6 of the 11 G6P[6] strains showed they had high homology with DS-1–like strains and clustered within the I2 genotype. However, 4 of these had high homology with the B1711 and other human strains belonging to the I2 genotype, and the other 2 shared high nucleotide identity with bovine strains, indicating circulation of 2 subsets of G6P[6] strains in Burkina Faso. Altogether, these findings indicate that animal or animal-derived rotavirus strains circulate in humans in Burkina Faso and that they emerged from different, independent reassortment events. A recent review suggested that 14% of African ROTAV strains had originated in whole or in part from an animal host (*13*).

To gain additional understanding of the evolution of these novel G6P[6] strains, we randomly selected 4 strains (3 with a likely human VP6, 1 with a bovine VP6 gene) and sequenced the nonstructural genes (NSP1–5). Genes NSP1–3 and NSP5 shared high homology with those of strain B1711, but relatively low homology was observed for the G6P[6] NSP4 gene, indicating a different human parental strain than that for B1711. The NSP4 gene shared high homology with the NSP4 gene of a G8P[6] strain (DRC86; 98.5% nt identity) and with a bovine ROTAV strain (UK; 96.3% nt identity). We classified the locally circulating G6P[6] strains as G6-P[6]-I2-A2-N2-T2-E2-H2, belonging to the DS-1 genogroup. We also attempted to sequence the VP1–3 genes, but only short sequence data for VP1 and VP2 could be obtained, which classified these genes as R2 and C2, respectively, also belonging to the DS-1 genogroup. Sequencing data for the genes (VP4, VP6, VP7, NSP1–5) indicated that the G6P[6] strains detected in Burkina Faso have evolved as a result of co-infection with locally circulating subgroup I specific P[6] strains and a bovine strain and/or human G6 strains. The result of these co-infections possibly resulted in generation of 2 types of G6P[6] strains, in 1 of which the VP6 gene is also of bovine origin. These G6P[6] strains highlight the need to further assess the efficacy of the currently licensed vaccines for such unusual and emerging genotypes.

To conclude, this study describes the diversity of ROTAV strains circulating in Ouagadougou, Burkina Faso, during 2009–2010 and a high incidence of the G6P[6] strain. We found a shift of VP6 G and P types in the middle of the ROTAV season and differences in clinical profiles; G9P[8]SGII strains induced more severe signs and symptoms than SGI strains, which infected older children. Moreover, we found bovine/human reassortant ROTAVs in children. The study highlights the need for continued monitoring of the detection assays and molecular surveillance of ROTAV strains in humans and animals in Burkina Faso. Such monitoring will identify strain diversity and emerging strains. There has not been much evaluation regarding the efficacy of the current rotavirus vaccines against rare ROTAV strains, such as G6P[6]; thus, information about strain diversity and emerging strains would also be useful during post-vaccine follow-up studies to determine vaccine efficacy.

## Supplementary Material

Technical Appendix GenBank accession numbers and genes of the rotavirus strains sequenced, Percent nucleotide identity of NSP1–5 of G6P[6] rotavirus strains from Burkina Faso with the prototype strains of each genotype, and GenBank accession numbers to reference rotavirus sequences in a study of the emergence of unusual G6P[6] rotaviruses in children with acute gastroenteritis, Burkina Faso, 2009–2010.
