# Supplementary material for: Emergence of Unusual G6P[6] Rotaviruses in Children, Burkina Faso, 2009–2010
Source: Emerg Infect Dis. 2012 Apr;18(4):589–97. doi: 10.3201/eid1804.110973 (PMC3309693; doi:10.3201/eid1804.110973)
Supplement: Technical Appendix — GenBank accession numbers and genes of the rotavirus strains sequenced, Percent nucleotide identity of NSP1–5 of G6P[6] rotavirus strains from Burkina Faso with the prototype strains of each genotype, and GenBank accession numbers to reference rotavirus sequences in a study of the emergence of unusual G6P[6] rotaviruses in children with acute gastroenteritis, Burkina Faso, 2009–2010. [file 11-0973-Techapp_6p.pdf]

Article DOI: http://dx.doi.org/10.3201/eid1804.110973

Emergence of Unusual G6P[6] Rotaviruses in Children, Burkina Faso, 2009–2010

Technical Appendix

Technical Appendix Table 1. GenBank accession numbers and genes of the rotavirus strains sequenced in a study of the emergence of unusual G6P[6] rotaviruses in children with acute gastroenteritis, Burkina Faso, 2009–2010

Strain	Gene	Accession no.
RVA/Human-wt/BFA/309-BF/2010/G3P[6]	VP7	JN116505
RVA/Human-wt/BFA/266-BF/2010/G3P[6]	VP7	JN116506
RVA/Human-wt/BFA/268-BF/2010/G3P[6]	VP7	JN116507
RVA/Human-wt/BFA/270-BF/2010/G3P[6]	VP7	JN116508
RVA/Human-wt/BFA/276-BF/2010/G3P[6]	VP7	JN116509
RVA/Human-wt/BFA/306-BF/2010/G3P[6]	VP7	JN116510
RVA/Human-wt/BFA/234-BF/2010/G6P[6]	VP4	JN116511
RVA/Human-wt/BFA/238-BF/2010/G6P[6]	VP4	JN116512
RVA/Human-wt/BFA/240-BF/2010/G6P[6]	VP4	JN116513
RVA/Human-wt/BFA/249-BF/2010/G6P[6]	VP4	JN116514
RVA/Human-wt/BFA/263-BF/2010/G6P[6]	VP4	JN116515
RVA/Human-wt/BFA/265-BF/2010/G6P[6]	VP4	JN116516
RVA/Human-wt/BFA/272-BF/2010/G6P[6]	VP4	JN116517
RVA/Human-wt/BFA/277-BF/2010/G6P[6]	VP4	JN116518
RVA/Human-wt/BFA/309-BF/2010/G3P[6]	VP4	JN116519
RVA/Human-wt/BFA/240-BF/2010/G6P[6]	VP6	JN116520
RVA/Human-wt/BFA/277-BF/2010/G6P[6]	VP6	JN116521
RVA/Human-wt/BFA/238-BF/2010/G6P[6]	VP6	JN116522
RVA/Human-wt/BFA/263-BF/2010/G6P[6]	VP6	JN116523
RVA/Human-wt/BFA/265-BF/2010/G6P[6]	VP6	JN116524
RVA/Human-wt/BFA/272-BF/2010/G6P[6]	VP6	JN116525
RVA/Human-wt/BFA/234-BF/2010/G6P[6]	VP7	JN116526
RVA/Human-wt/BFA/238-BF/2010/G6P[6]	VP7	JN116527
RVA/Human-wt/BFA/240-BF/2010/G6P[6]	VP7	JN116528
RVA/Human-wt/BFA/249-BF/2010/G6P[6]	VP7	JN116529
RVA/Human-wt/BFA/263-BF/2010/G6P[6]	VP7	JN116530
RVA/Human-wt/BFA/265-BF/2010/G6P[6]	VP7	JN116531
RVA/Human-wt/BFA/272-BF/2010/G6P[6]	VP7	JN116532
RVA/Human-wt/BFA/277-BF/2010/G6P[6]	VP7	JN116533
RVA/Human-wt/BFA/285-BF/2010/G6P[6]	VP7	JN116534
RVA/Human-wt/BFA/288-BF/2010/G6P[6]	VP7	JN116535
RVA/Human-wt/BFA/307-BF/2010/G6P[6]	VP7	JN116536
RVA/Human-wt/BFA/238-BF/2010/G6P[6]	NSP1	JN116537
RVA/Human-wt/BFA/263-BF/2010/G6P[6]	NSP1	JN116538
RVA/Human-wt/BFA/265-BF/2010/G6P[6]	NSP1	JN116539
RVA/Human-wt/BFA/272-BF/2010/G6P[6]	NSP1	JN116540
RVA/Human-wt/BFA/238-BF/2010/G6P[6]	NSP2	JN116541
RVA/Human-wt/BFA/263-BF/2010/G6P[6]	NSP2	JN116542
RVA/Human-wt/BFA/265-BF/2010/G6P[6]	NSP2	JN116543
RVA/Human-wt/BFA/272-BF/2010/G6P[6]	NSP2	JN116544
RVA/Human-wt/BFA/238-BF/2010/G6P[6]	NSP3	JN116545
RVA/Human-wt/BFA/263-BF/2010/G6P[6]	NSP3	JN116546
RVA/Human-wt/BFA/265-BF/2010/G6P[6]	NSP3	JN116547
RVA/Human-wt/BFA/272-BF/2010/G6P[6]	NSP3	JN116548
RVA/Human-wt/BFA/238-BF/2010/G6P[6]	NSP4	JN116549
RVA/Human-wt/BFA/263-BF/2010/G6P[6]	NSP4	JN116550
RVA/Human-wt/BFA/265-BF/2010/G6P[6]	NSP4	JN116551
RVA/Human-wt/BFA/272-BF/2010/G6P[6]	NSP4	JN116552
RVA/Human-wt/BFA/238-BF/2010/G6P[6]	NSP5	JN116553
RVA/Human-wt/BFA/263-BF/2010/G6P[6]	NSP5	JN116554

Strain	Gene	Accession no.
RVA/Human-wt/BFA/265-BF/2010/G6P[6]	NSP5	JN116555
RVA/Human-wt/BFA/272-BF/2010/G6P[6]	NSP5	JN116556
RVA/Human-wt/BFA/225-BF/2010/G1P[6]	VP4	JQ255029
RVA/Human-wt/BFA/259-BF/2010/G1P[6]	VP4	JQ255030
RVA/Human-wt/BFA/267-BF/2010/G1P[6]	VP4	JQ255031
RVA/Human-wt/BFA/268-BF/2010/G3P[6]	VP4	JQ255032
RVA/Human-wt/BFA/281-BF/2010/G1G6P[6]	VP4	JQ255033

Technical Appendix Table 2. Percent nucleotide identity of NSP1–5 genes of G6P[6] rotavirus strains from Burkina Faso, December 2009–March 2010, with the prototype strains of each genotype*

NSP1 (nt 215-1352)†			NSP2 (nt 88-923)‡			NSP3 (nt 247-946)§			NSP4 (nt 35-721)¶			NSP5 (nt 68-624)‖		
Genotype, strain	G and P genotype	% nt identity	Genotype, strain	GxP[y]	% nt identity	Genotype, strain	GxP[y]	% nt identity	Genotype, strain	GxP[y]	% nt identity	Genotype, strain	GxP[y]	% nt identity
A1, Wa	G1P[8]	75.4	N1, Wa	G1P[8]	81.3	T1, Wa	G1P[8]	76.1	E1 Wa	G1P[8]	82.4	H1, Wa	G1P[8]	83.9
A1, KU	G1P[8]	75.0	N1, KU	G1P[8]	82.1	T1, KU	G1P[8]	75.8	E1 KU	G1P[8]	82.3	H1, KU	G1P[8]	83.9
A2, DS1	G2P[4]	93.0	N2, DS1	G2P[4]	86.8	T2, DS1	G2P[4]	94.6	E2 DS1	G2P[4]	90.7	H2, DS1	G2P[4]	95.6
**A2**, **DRC86**	**G8P[6]**	**97.1**	**N2, DRC86**	**G8P[6]**	**97.8**	**T2, DRC86**	**G8P[6]**	**97.8**	**E2 DRC86**	**G8P[6]**	**98.5**	**H2, DRC86**	**G8P[6]**	**97.6**
**A2**, **B1711**	**G6P[6]**	**97.6**	**N2, B1711**	**G6P[6]**	**99.5**	**T2, B1711**	**G6P[6]**	**97.5**	E2 B1711	G6P[6]	90.1	**H2, B1711**	**G6P[6]**	**98.3**
**A2**, **RV176-00**	**G12P[6]**	**97.5**	**N2, RV176-00**	**G12P[6]**	**97.1**	**T2, RV176-00**	**G12P[6]**	**97.8**	E2 TB-Chen	G2P[4]	89.9	**H2, RV176-00**	**G12P[6]**	**98.0**
A3, *BoRF*	G6P[1]	64.2	N2, *BoRF*	G6P[1]	86.0	T3, Au-1	G3P[9]	80.9	E2 *BoRF*	G6P[1]	89.0	H3, *BoRF*	G6P[1]	85.1
A3, *BoUK*	G6P[5]	64.2	N2, *BoUK*	G6P[5]	87.8	T4, PO-13	G18P[17]	55.8	**E2 *BoUK***	**G6P[5]**	**96.3**	H3, *BoUK*	G6P[5]	83.8
A3, *BoWC3*	G6P[5]	64.3	N2, *BoWC3*	G6P[5]	86.8	T5, SA11-5N	G3P[1]	75.3	E2 *BoWC3*	G6P[5]	87.6	H3, *BoWC3*	G6P[5]	85.8
A3, Au-1	G3P[9]	63.4	N3, Au-1	G3P[9]	78.7	T6, OVR762	G8P[14]	76.4	E3 Au-1	G3P[9]	81.3	H3, Au-1	G3P[9]	83.9
A4, PO-13	G18P[17]	42.0	N4, PO-13	G18P[17]	62.5	T6, *BoRF*	G6P[1]	76.5	E4 Ty-1	G7P[17]	55.5	H4, PO-13	G18P[17]	66.2
A5, SA11-H96	G3P[2]	52.2	N5, SA11-5N	G3P[1]	77.4	T6, *BoWC3*	G6P[5]	76.1	E5 ALA	G3P[14]	84.1	H5, SA11-5N	G3P[1]	84.9
A6, L338	G13P[18]	48.6				T7, *BoUK*	G6P[5]	75.0	E6 RV176-00	G12P[6]	81.7	H6, RRV	G3P[3]	83.6
A7, EW	G16P[16]	47.2							E7, EW	G16P[16]	69.7			
A8, Gottfried	G4P[6]	71.9							E8, CMP034	G2P[27]	79.7			
A9, ALA	G3P[14]	49.6												
A10, FI14	G3P[12]	49.5												
A11, OVR762	G8P[14]	64.1												
A12, T152	G12P[9]	59.9												
A13, B223	G10P[11]	64.7												
A14, A5-13	G8P[1]	66.7												

*__Boldface__ indicates high nucleotide similarity with reference strain; *italic* font indicates bovine G6 strains. NSP, nonstructural protein.
†Position corresponding to the B1711 strain. GenBank accession numbers for reference strains are available in Technical Appendix Table 6.
‡GenBank accession numbers for reference strains are available in Technical Appendix Table 7.
§GenBank accession numbers for reference strains are available in Technical Appendix Table 8.
¶GenBank accession numbers for reference strains are available in Technical Appendix Table 9.
‖GenBank accession numbers for reference strains are available in Technical Appendix Table 10

Technical Appendix Table 3. Accession numbers to VP4 genes of reference strains in Figure 3

Strain	Accession no.
R308	EU033985
B1711	EF554085
NB287	DQ070467
6818NN	AF529876
Dhaka107	DQ492649
SL273	HM136999
DRC88	DQ005111
DRC86	DQ005122
86/Ghana	AY843335
5001DB/97	AF529873
134/04-8	AY955301
221/04-7	AY955303
1076	M88480
6342LP/99	AF529874
BP720/93	AJ621503
BP1198/98	AJ621504
BP1338/99	AJ621507
Ch9	AF183861
Gottfried	M33516
JP3-6	AB176685
JP29-6	AB176688
M37	L20877
MtA5	L25268
MW23	AJ278253
NB123/86	AF161828
P14-3	EU348717
P83	EU348718
RV3	U16299
SI-MB6	EU348716
US1205	AF079356

Technical Appendix Table 4. Accession numbers to VP7 genes of reference strains in Figure 4, panel A

Strain	Accession no.
B1711	EF554087
R353	DQ122400
Hun7	AJ488134
10733	AY281360
CIT-A99	GQ377868
RVL-Bov4	GQ433987
Se584	EF672609
DRC88	DQ005109
WC3	AY050272
PA151	L20881

Technical Appendix Table 5. Accession numbers to VP6 genes of reference strains in Figure 4, panel B

Strain	Accession no.
Wa	K02086
KU	AB022768
Dhaka16-03	DQ492673
D	EF583024
DS-1	EF583028
TB-chen	AY787645
P	EF583040
AU-1	DQ490538
ST3	EF583048
IAL28	EF583032
Se584	EF583044
DRC86	DQ005121
DRC88	DQ005110
WI61	EF583052
L26	EF583036
Dhaka12-03	DQ146664
Matlab13-03	DQ146675
N26-02	DQ146686
RV176-00	DQ490555
RV161-00	DQ490549
B4633-03	DQ146642
Dhaka25-02	DQ146653
T152	DQ146702
B1711	EF554086
HP140	DQ003295
RUBV81	EF200566
86	GU984759
Bo/Ind/UKD/09/M-1	HM235508
WC3	AF411322

Technical Appendix Table 6. Accession numbers to NSP1 genes of reference strains in Technical Appendix Table 2

Strain	Accession no.
Wa	L18943
KU	AB022769
DS1	L18945
DRC86	DQ005119
B1711	EF554088
RV176-00	DQ490557
RF	M22308
UK	HQ186289
WC3	EF990699
Au-1	D45244
PO-13	AB009633
SA11-H96	DQ838599
L338	D38158
EW	U08428

Technical Appendix Table 7. Accession numbers to NSP2 genes of reference strains in Technical Appendix Table 2

Strain	Accession no.
Wa	L04534
KU	AB022770
DS1	L04529
DRC86	DQ005118
B1711	EF554089
RV176-00	DQ490558
RF	Z21640
UK	J02420
WC3	EF990700
Au-1	DQ490534
PO-13	AB009625
SA11-5N	DQ838611

Technical Appendix Table 8. Accession numbers to NSP3 genes of reference strains in Technical Appendix Table 2

Strain	Accession no.
Wa	X81434
KU	AB022771
DS1	EF136660
DRC86	DQ005117
B1711	EF554090
RV176-00	DQ490559
Au-1	DQ490535
PO-13	AB009626
SA11-5N	DQ838606
OVR762	EF554156
RF	Z21639
WC3	EF990701
UK	K02170

Technical Appendix Table 9. Accession numbers to NSP4 genes of reference strains in Technical Appendix Table 2

Strain	Accession no.
Wa	AF093199
KU	AB022772
DS1	AF174305
DRC86	DQ005116
B1711	EF554091
TB-Chen	AY787650
RF	AY116593
UK	K03384
WC3	AY050273
Au-1	D89873
Ty-1	AB065285
ALA	AF144792
RV176-00	DQ490560
EW	U96335

Technical Appendix Table 10. Accession numbers to NSP5 genes of reference strains in Technical Appendix Table 2

Strain	Accession no.
Wa	V01191
KU	AB008661
DS1	M33608
DRC86	DQ005115
B1711	EF554092
RV176-00	DQ490561
RF	AF188126
UK	K03385
WC3	EF990702
Au-1	AB008656
PO-13	AB009628
SA11-5N	DQ838626
RRV	HQ665467
